# Alternating Superconducting and Charge Density Wave Monolayers within Bulk 6R-TaS_2_

**DOI:** 10.1021/acs.nanolett.2c01851

**Published:** 2022-07-20

**Authors:** Amritroop Achari, Jonas Bekaert, Vishnu Sreepal, Andrey Orekhov, Piranavan Kumaravadivel, Minsoo Kim, Nicolas Gauquelin, Premlal Balakrishna Pillai, Johan Verbeeck, Francois M. Peeters, Andre K. Geim, Milorad V. Milošević, Rahul R. Nair

**Affiliations:** † National Graphene Institute, University of Manchester, Manchester M13 9PL, United Kingdom; ‡ Department of Chemical Engineering, University of Manchester, Manchester M13 9PL, United Kingdom; § Department of Physics, University of Antwerp, Groenenborgerlaan 171, B-2020, Antwerp, Belgium; ∥ NANOlab Center of Excellence, University of Antwerp, Groenenborgerlaan 171, B-2020 Antwerp, Belgium; ⊥ Electron Microscopy for Materials Science (EMAT), University of Antwerp, Groenenborgerlaan 171, B-2020 Antwerp, Belgium; # Department of Physics and Astronomy, University of Manchester, Manchester M13 9PL, United Kingdom

**Keywords:** 2D materials, Superconductivity, Bulk van der Waals heterostructure, Charge density waves, TaS_2_

## Abstract

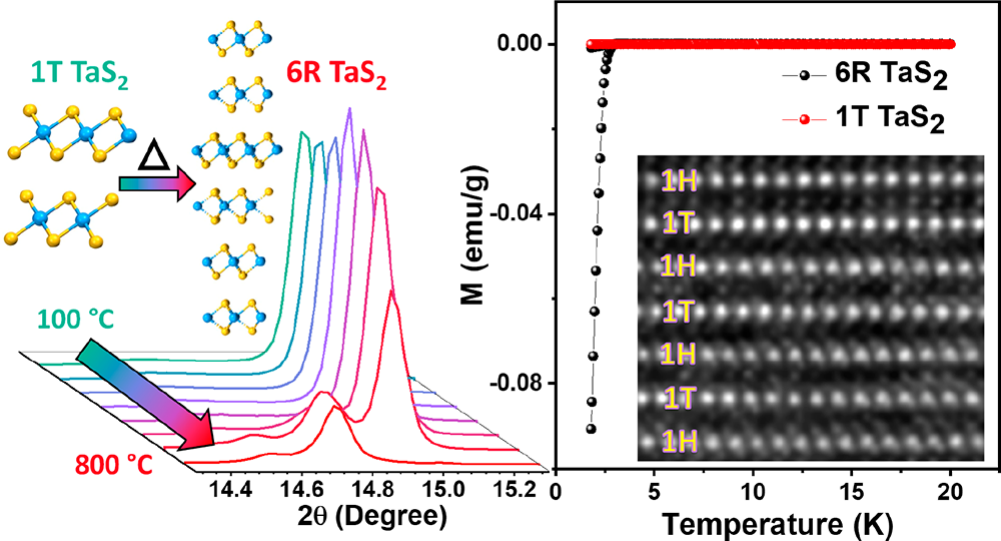

Van der Waals (vdW) heterostructures continue to attract intense interest as a route of designing materials with novel properties that cannot be found in nature. Unfortunately, this approach is currently limited to only a few layers that can be stacked on top of each other. Here, we report a bulk vdW material consisting of superconducting 1H TaS_2_ monolayers interlayered with 1T TaS_2_ monolayers displaying charge density waves (CDW). This bulk vdW heterostructure is created by phase transition of 1T-TaS_2_ to 6R at 800 °C in an inert atmosphere. Its superconducting transition (*T*_c_) is found at 2.6 K, exceeding the *T*_c_ of the bulk 2H phase. Using first-principles calculations, we argue that the coexistence of superconductivity and CDW within 6R-TaS_2_ stems from amalgamation of the properties of adjacent 1H and 1T monolayers, where the former dominates the superconducting state and the latter the CDW behavior.

Designing heterostructured materials with tailor-made properties has significant importance in fundamental and applied research. For example, the development of III–V semiconductor heterostructures^1^ has transformed many aspects of our lives. More recently, the fabrication of heterostructures using two-dimensional (2D) materials with complementary properties^2^ opens an astounding number of opportunities for designing exotic materials. Since 2D materials come in a plethora of different physical, chemical, and electronic properties, the number of possible combinations we can achieve is unlimited, paving the way for materials with tailor-made properties. The layers in 2D heterostructures are held by their van der Waals interaction and are commonly referred to as van der Waals (vdW) heterostructures. They have already shown promise in different applications. For example, vdW heterostructures made using metal–insulator, metal–semiconductor, and insulator–insulator 2D materials not only exhibit new physics (e.g., Hofstader butterfly states in graphene/hBN,^3^ ultrafast charge transfer in MoS_2_/WS_2_ interface^4^) but show good performance in electronic and optoelectric applications such as field-effect transistors,^5−7^ photodetectors^8−10^ and light-emitting diodes.^11,12^ In addition, some interesting properties of transition metal dichalcogenides such as 2D superconductivity^13^ and charge density wave states^14^ can also be tuned through interlayer coupling via vdW heterostructures.

Most of the vdW heterostructures are currently prepared by mechanically stacking one 2D layer on another. Even though this process is laborious, the precision in creating the heterostructure makes this process ideal for probing the fundamental properties of the heterostructure devices. On the other hand, the direct synthesis of vdW heterostructures in the chemical vapor deposition (CVD) process or via chemical methods is far from perfect. It is noteworthy that vdW heterostructure in bulk also exists in nature. Frankencite is a natural vdW heterostructure formed by alternate stacking of SnS_2_ and PbS layers.^15^ Examples of such bulk vdW heterostructures are rare, and even with the progress in the 2D materials research, synthesizing such bulk vdW heterostructures are still challenging. The 6R phase of TaS_2_ with alternating layers of 1H (superconducting)^16,17^ and 1T TaS_2_ (Mott insulator)^18^ is another example of bulk vdW heterostructure. This phase has rarely been studied due to the difficulty and inconsistency in synthesizing the pure phase via the traditional vapor transport method.^19,20^ In this work, we report interlayered monolayer superconductivity and charge density waves (CDW) in 6R TaS_2_ obtained by a thermally driven phase conversion of 1T TaS_2_. We establish the bulk heterostructure of 1H and 1T layers in 6R phase by electron microscopy. We also show that superconductivity and CDW coexist in this bulk vdW heterostructure, and the superconducting transition temperature (2.6 K) is higher than that in both 2H (0.8 K)^16^ and 1T phases of TaS_2_ (1.5 K at 2.5 GPa).^18^ Through exfoliation and restacking, we have demonstrated that the superconducting transition temperature can be further increased to 3.6 K.

Figure 1a shows the *in situ* temperature-dependent evolution of the (001) XRD peak of a single crystalline 1T TaS_2_ under high vacuum. At temperatures below 500 °C, we only observed a peak shift associated with the thermal expansion of the crystalline *c*-axis (Figure S1). But at 600 °C we noticed the appearance of a new peak at lower 2θ (14.78°) compared to the original peak of the 1T phase at 15.05°. Further increase in temperature resulted in steady decrease in 1T peak intensity with a concurrent increase in the intensity of the new peak at 14.78°. At 800 °C, no trace of the 1T peak remained in the XRD pattern. Since the sample was single-crystalline and highly oriented in nature, we only observed (*00l*) peaks in the XRD pattern. To fully understand the structural transformation after annealing, we performed further powder X-ray diffraction (PXRD) studies after grinding the 800 °C annealed crystal in a mortar. The resulting PXRD pattern shows peaks associated with other lattice planes in addition to (*00l*) peaks (Figure 1b). It is to be noted that the intensity of the peaks associated with the (*00l*) planes is higher due to the incomplete grinding. Upon matching the diffraction pattern to that of powdered 1T and simulated PXRD patterns from CIF files of 1T, 2H and 6R TaS_2_, we found that it matches exclusively with 6R phase^21^ (Figure S2). Figure 1b inset shows the zoomed view of the low angle peak and its comparison to the corresponding calculated peak positions of parent 1T^22^ (blue line), 2H^23^ (green line), and 6R (red line) phases of TaS_2_. This further confirms that the 1T TaS_2_ samples undergo phase transition while annealing in a vacuum. We have also performed control experiments where single-crystalline 2H TaS_2_ was heated up to 800 °C. *In situ* XRD does not show any phase change (Figure S3), suggesting only 1T phase can be converted into 6R phase by annealing.

**Figure 1:**
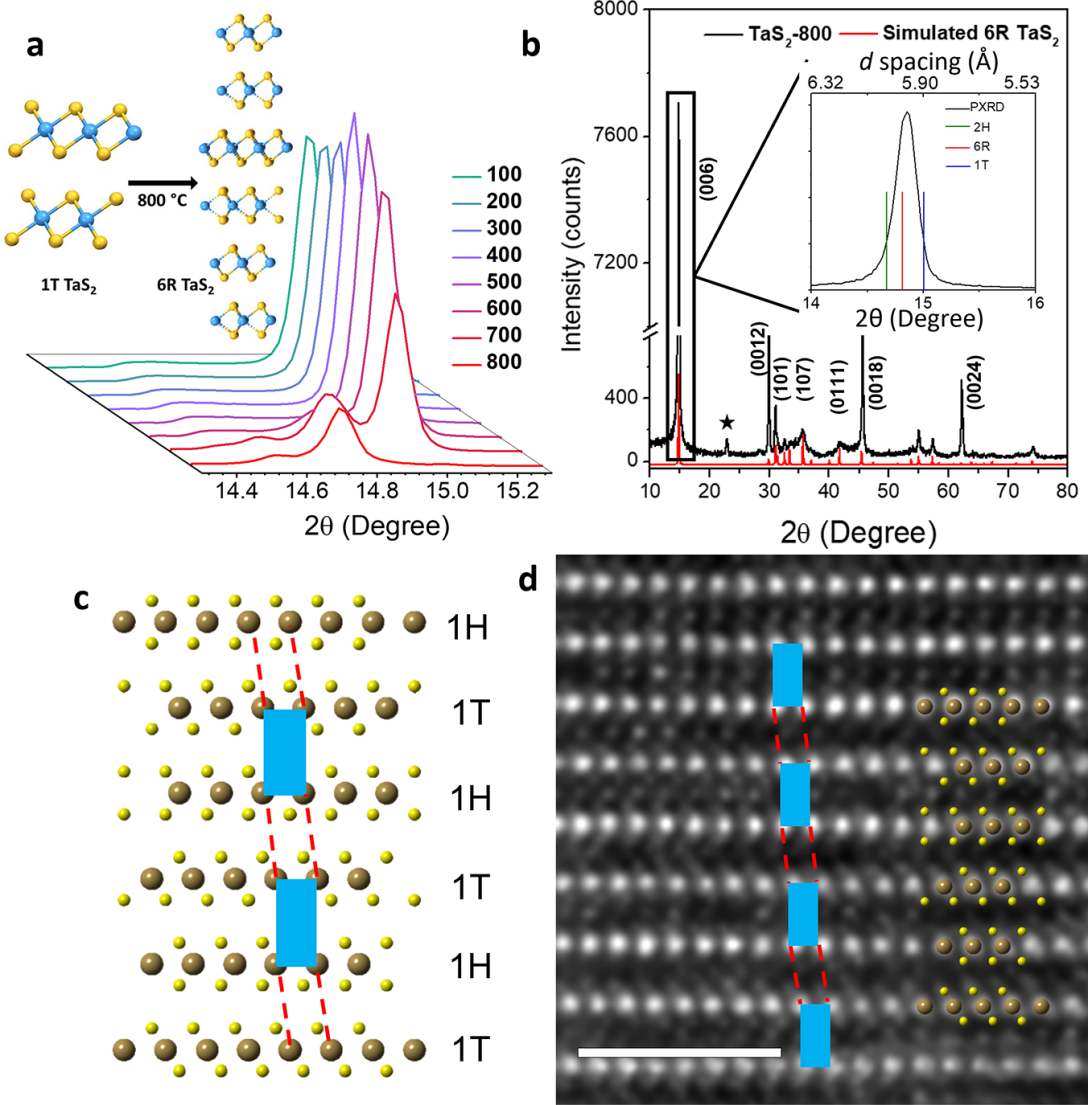
The 1T to 6R phase transition of TaS_2_. (a) *In situ* temperature-dependent XRD of a 1T TaS_2_ single-crystal heated in vacuum up to 800 °C. Inset: Schematic of phase transition of 1T TaS_2_ to 6R phase. The teal spheres represent Ta and the yellow spheres represent S atoms. (b) PXRD pattern of the powdered form of 800 °C heated 1T crystal (black) compared to 6R phase reference spectra (red) by using the model.^21^ * indicates peak from surface oxidation due to the residual air in the vacuum chamber. Inset: Zoomed-in view of the (006) peak shown inside the rectangle. Green, red, and blue lines show the (*00l*) peak position corresponding to 2H, 6R, and 1T phases of TaS_2_, respectively. (c) Model crystal structure of 6R TaS_2_ showing alternating layers of 1T and 1H TaS_2_. Blue rectangle and red dotted lines show each 1T-1H hetero layer are slightly displaced in the *c*-axis. (d) Cross-sectional high-resolution STEM image of annealed TaS_2_ sample along [110] direction showing the alternating arrangement of 1H and 1T layers. Scale bar, 2 nm. Overlaying 6R atomic model structure shows match of atomic positions and lattice stacking with the STEM image. In the model, Ta atoms are denoted as brown and S atoms as yellow spheres. The blue rectangle and the red dotted lines show that, similar to the model structure, each 1T-1H hetero layer is slightly displaced in the *c*-axis.

The lower angle (006) XRD peak in 6R TaS_2_ suggests a lattice expansion in the *c*-direction after the phase change. The interlayer spacing corresponding to the (006) peak estimated from the XRD pattern was 0.597 ± 0.001 nm for the 6R phase. On the other hand, the estimated interlayer spacing of the parent 1T phase was 0.590 nm, marking a 1.4% lattice expansion along the *c*-direction.

We have performed cross-sectional high-resolution scanning transmission electron microscopy (HRSTEM) of the annealed sample to confirm the phase transition into the 6R phase. Figure 1d shows a STEM image of the annealed sample at room temperature showing alternate stacks of 1T and 1H layers. We found a perfect match of atomic positions and lattice stacking of the annealed sample with a model 6R structure, ruling out the presence of any other polytype of TaS_2_. Further, the interlayer spacing estimated from the intensity profiles (Figure S4) for the 1T to 6R phase (before and after heating) showed a 1.6% increase from 0.596 ± 0.006 nm to 0.606 ± 0.006 nm (Figure S5), indicating a transition to the 6R phase and closely matching the PXRD value. Additionally, the Raman spectra of bulk 6R sample shows the presence of Raman active modes from both 1T and 2H phases, (Figure S6) confirming its heterobilayer structure.

Subsequently, we have investigated the electrical and magnetic properties of bulk 6R TaS_2_. Figure 2a shows typical magnetization versus temperature curves, *M*(*T*), for 6R TaS_2_ and parent 1T TaS_2_ crystal under the external magnetic field of 5 Oe. Zero field cooling (ZFC) data for 6R TaS_2_ clearly shows a diamagnetic transition at ∼2.5 K (shielding of the external field, *H*, which is characteristic of superconducting materials). The onset temperature of this superconducting transition is much higher than the transition temperature (*T*_c_) of 2H phase (0.8 K). In comparison, the parent 1T phase or 1T TaS_2_ annealed at different temperatures up to 600 °C does not show any diamagnetic transition as expected (Figure 2a and Figure S7). We have also carried out magnetic measurements on 2H TaS_2_, and 2H TaS_2_ heated at 800 °C, but no transition down to 1.8 K was observed (Figure S8), ruling out the presence of impurities or defects as a cause of the superconducting transition in the heated 1T sample. FC-ZFC measurements performed at higher fields reveal that the onset temperature of superconductivity (*T*_c_) decreases when *H* is increased (Figure 2a inset). No onset of superconductivity was observed at fields higher than 500 Oe. Upon exceeding this field, the samples show only a weak paramagnetic signal. The *M–H* curve in Figure 2b exhibits a typical magnetic hysteresis profile of a type II superconductor. The phase diagram (Figure 2b inset) was obtained by calculating *H*_C2_ at different temperatures from the divergence point on the *X*-axis. From the phase diagram, the *T*_c_ of the material was estimated to be 2.6 K. The obtained 6R TaS_2_ was stable in air. No change in magnetic measurements was observed after exposing the sample to an ambient atmosphere for one month.

**Figure 2:**
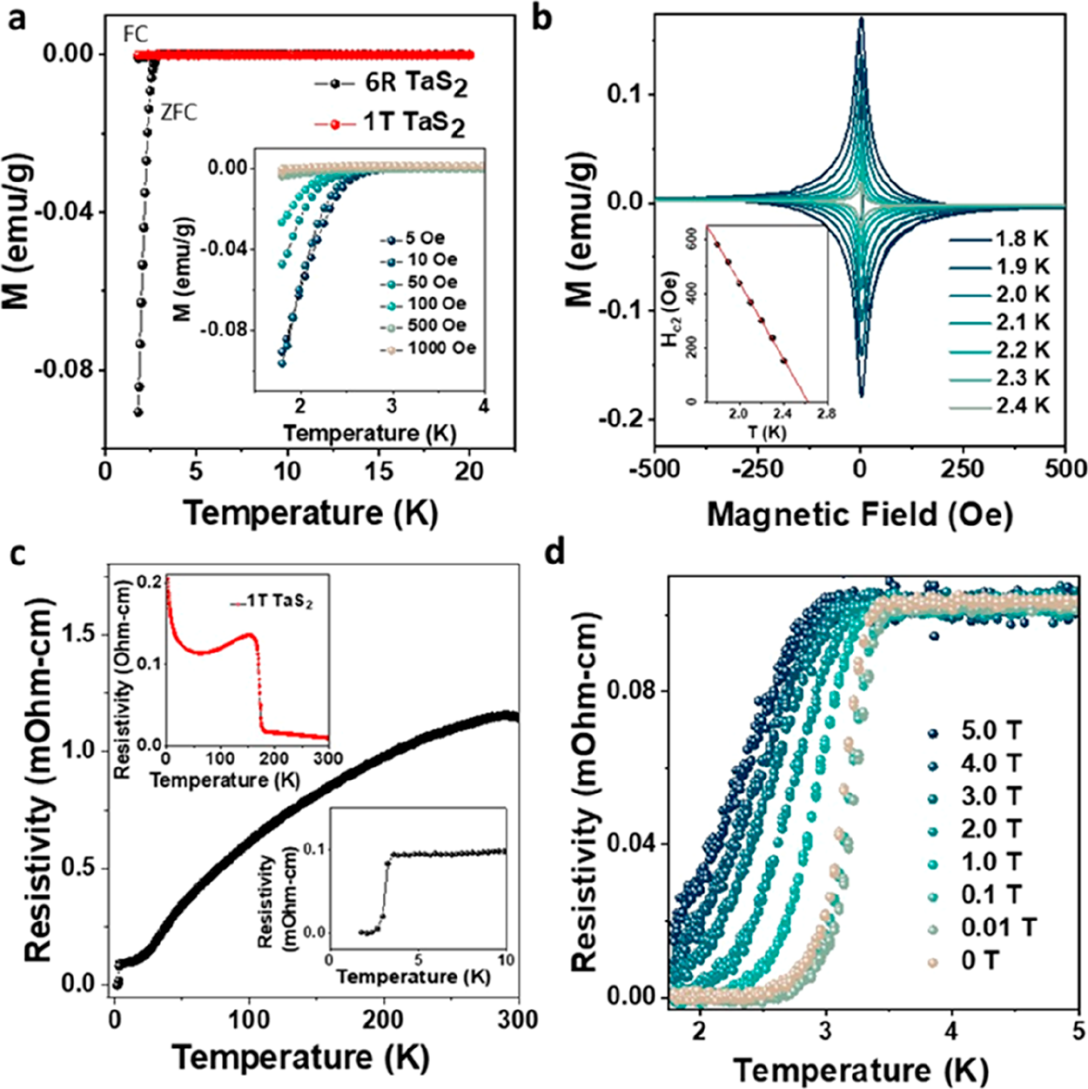
Superconductivity in 6R TaS_2_. (a) Temperature dependence of ZFC and FC magnetization, M, for single-crystalline 6R TaS_2_ and 1T TaS_2_ under the magnetic field of 5 Oe applied parallel to the *c*-axis. The inset shows ZFC and FC *M*(*T*) at different magnetic fields for 6R TaS_2_. (b) Magnetisation dependence as a function of *H* ∥ *c* at different temperatures. The inset shows the temperature dependence of the upper critical field *H*_C2_. The upper critical field was calculated from the divergence point in the *M–H* hysteresis curve. The solid red line is the guide to the eye. (c) Temperature dependence of electrical resistivity of 6R TaS_2_ crystal at *H* = 0 T. Bottom inset shows zoomed superconducting transition. The top inset shows the temperature dependence of electrical resistivity of 1T TaS_2_ nanosheets at *H* = 0 T. (d) The evolution of *R*(*T*) for 6R TaS_2_ with increasing external magnetic field in an *H* ∥ *ab* geometry.

With TaS_2_ being a layered material, we expect its superconductivity to be anisotropic across different crystalline directions. To investigate this, we studied the dependence of magnetic field orientation on the observed transition. All of the magnetization studies so far were performed with a geometry where the *c*-axis of the TaS_2_ crystal lies parallel to the magnetic field orientation (*H* ∥ *c*). Upon rotating the crystal plane orientation such that *H* ∥ *ab*, the observed diamagnetic transition is almost negligible (Figure S9a). Further, we have calculated the temperature dependence of critical magnetic field (*H*_C2_) for parallel and perpendicular geometries and found the magnetic anisotropy (*H*_*C*2_^⊥^)/(*H*_C2_^∥^) to be 40 (Figure S9b). Such a high anisotropy indicates the 2D nature of superconductivity in 6R TaS_2_. This value closely resembles the anisotropy observed in intercalated 2H TaS_2_ (47),^24^ and is much higher than other TaS_2_ based systems such as 2H TaS_2_ (6.7),^24^ Pb_1/3_TaS_2_ (17),^25^ restacked TaS_2_ (11)^26^ and 4Hb TaS_2_ (17).^27^

Further evidence for superconductivity in 6R TaS_2_ crystal was obtained from low-temperature electrical resistivity measurements. Figure 2c shows the zero-field resistivity of 6R TaS_2_ plotted against temperature for the current flowing in the *ab* plane. At high temperatures, the resistivity decreases almost linearly with temperature, showing the semimetallic nature of 6R TaS_2_. Upon lowering the temperature, a superconducting transition (where resistivity reaches zero) is observed at 2.6 K (Figure 2c bottom inset) in agreement with our magnetic measurements. On the other hand, the parent 1T TaS_2_ (Figure 2c top inset) does not show any superconducting transition, but a large charge density wave (CDW) transition at 180 K from nearly commensurate CDW to commensurate CDW was observed as reported previously.^28,29^ Field-dependent resistivity measurements on 6R TaS_2_ were performed with the magnetic field parallel to the *ab* plane of the sample (*H* ∥ *ab*) and a decrease in superconducting transition temperature was observed with increasing field (Figure 2d).

Among TMDs, TaS_2_ has a unique place due to the exciting interplay between CDWs and superconductivity. Both 1T and 2H forms of TaS_2_ show CDWs,^29,30^ which are in direct competition with superconducting pairing. To probe the CDW in 6R TaS_2_, we have performed further electrical measurements above room temperature (Figure 3a). These measurements showed two closely spaced resistance transitions at 320 and 305 K. Similar transitions (350 and 180 K) were also observed in IT TaS_2_ and were attributed to the transition from the incommensurate (IC) CDW phase to nearly commensurate (NC) CDW phase and from nearly commensurate to the commensurate CDW (CCDW) phase, respectively.^31^ In the low-temperature commensurate phase, the Ta atoms of the 1T layers displace to form a commensurate superstructure with 13 Ta atoms arranged in the shape of star of David as depicted in the schematic in Figure 3a. The NC structure also possesses such atomic arrangement but in distant domains separated by a discommensuration network.^31^ To further probe the CDWs in 6R TaS_2_, we performed systemic temperature-dependent TEM studies from room temperature to 103 K. Figure 3b shows the selected area electron diffraction pattern from 6R TaS_2_ at room temperature (293 K). The additional diffraction spots (yellow circles in Figure 3b inset) show the presence of CDW. Similar spots were also observed in 1T TaS_2_ but only below 180 K^31^ suggesting the observed 305 K transition in 6R TaS_2_ is to a commensurate phase. We have also performed a temperature-dependent cryogenic electron diffraction study of the samples down to 103 K and observed no appearance of additional spots in the diffraction pattern of the sample (Figure S10). A room-temperature HRSTEM image of 6R TaS_2_ crystal shows a hexagonal arrangement of Ta atoms (Figure 3c inset). Fourier transformation of the image shows the appearance of six singlet spots in the first order *q* positions (marked with yellow arrows, Figure 3c). The presence of singlet spots further confirms the existence of the commensurate phase in 6R TaS_2_ at room temperature rather than NC phase, where the spots in first-order *q* positions appear as triplets.^32^ Raman spectra of bulk 6R sample also confirm the presence of commensurate structure in the 1T planes at room temperature (Figure S6). Further high-temperature electron diffraction or scanning tunnelling microscopy studies are required to fully confirm the nature of transition observed at 320 K. However, based on the 1T TaS_2_ CDWs, we attribute the transition at 320 K to the IC CDW phase to NC CDW phase.

**Figure 3:**
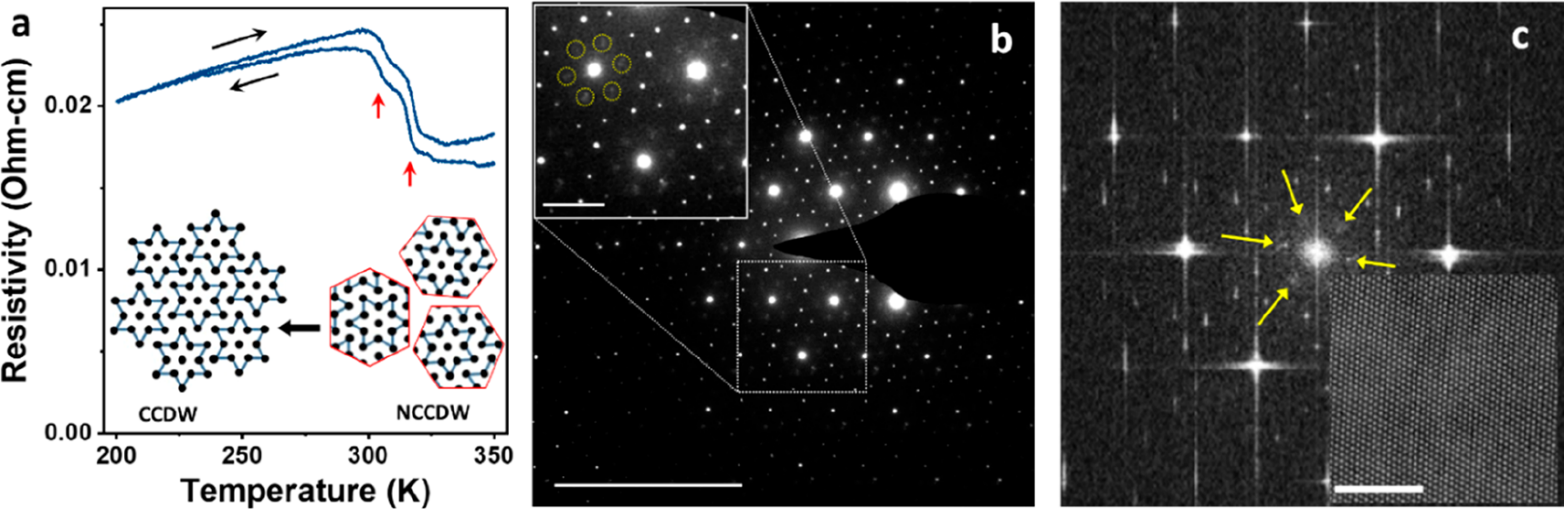
Charge density wave (CDW) in 6R TaS_2_. (a) Temperature-dependence of electrical resistivity of 6R TaS_2_ showing CDW transitions (red arrows). Black arrows denote temperature sweep direction. Schematic representation of phase transition from nearly commensurate to commensurate structure is shown below. (b) Selected area electron diffraction pattern from 6R TaS_2_ at room temperature (293 K). Scale bar, 10 1/nm. Top inset: zoomed view of the electron diffraction pattern from the white square marked area clearly showing CDW spots (yellow circles). Scale bar, 2 1/nm. (c) Fourier transformation of the High-resolution HAADF STEM image of 6R TaS_2_ (inset, scale bar, 5 nm) shows low-frequency spots (yellow arrows), suggesting a commensurate phase in the crystal.

One very interesting aspect of layered materials is the ability to separate them into single layer forms by chemical or physical exfoliation processes. To understand the effect of the reduced dimensionality on the superconductivity of 6R TaS_2_, we have exfoliated the as-prepared 6R TaS_2_ single crystal by Li intercalation (Supporting Information, Figure S11) followed by liquid-phase exfoliation using ultrasonication. The Li intercalated TaS_2_ was also found to be superconducting but with an enhanced *T*_c_ of 3.0 K (Figure S12). The liquid exfoliated layers in 6R TaS_2_ were then restacked (well separated and electronically decoupled) to obtain random stacking of layers in 6R TaS_2_ (Supporting Information). It is to be noted that 6R TaS_2_ consists of alternating 1H and 1T planes that separate from each other during exfoliation. Magnetic and transport measurements on the restacked 6R TaS_2_ showed an increased superconducting *T*_c_ of 3.6 K (Figure S13a–c). We observe a huge shift in CDW transition temperature in restacked 6R phase from NC to commensurate phase to 250 K from 320 K in bulk 6R TaS_2_ (Figure S13d), switching closer to the transition temperature observed in the 1T TaS_2_. Superconducting *T*_c_ and CDW *T*_c_ of restacked TaS_2_ samples closely resemble that of 1H and 1T layers, respectively. This indicates that 1T and 1H layers in 6R TaS_2_ are separated into 2H and 1T phases during exfoliation and restacking. We have also studied the superconducting *T*_c_ of mechanically exfoliated thin layers (∼1 nm) of 6R TaS_2_ and found its *T*_c_ similar to the bulk 6R TaS_2_ (Supporting Information, Figure S14).

To explain the underlying mechanisms responsible for the emergence of superconductivity and CDWs in 6R TaS_2_, we have performed first-principles calculations of its electronic and phononic properties, as well as the electron–phonon coupling and the resulting superconducting state (Supporting Information). The calculations were performed using density functional (perturbation) theory DFPT, as implemented in the ABINIT package.^33^ We started our investigation from the 1T and 1H monolayers (MLs), and the 1T-1H bilayer (BL), to establish a thorough bottom-up comparison between the elementary TMD phases and heterogeneous phases like the 1T-1H BL. The 6R TaS_2_ is composed of three such 1T-1H BLs arranged with rhombohedral stacking (see Figure 1c).

We focus first on the electronic properties of the different TaS_2_ structures around the Fermi level, directly relevant for their superconducting and CDW properties. The Fermi surface of the 1T-1H BL (Figure S15f) is essentially a combination of the individual Fermi surfaces of the two MLs (Figure S15d,e) with similar Fermi surface shapes and corresponding Fermi velocities (*v*_F_).

Nevertheless, there are some interesting effects of interlayer interactions. First, an avoided crossing occurs along the Γ–M direction, between the 1T- and 1H-based Fermi sheets (the former positioned around M, the latter around Γ and K). Second, there is a spin–orbit coupling (SOC)-induced splitting in the 1T-based sheet due to the lack of inversion symmetry in the BL originating from the 1H layer. The Fermi surface of the bulk 6R phase (Figure 4a) clearly is the 3D counterpart of the Fermi surface of the BL (Figure S15f) with nearly identical Fermi sheets and Fermi velocity distribution.

**Figure 4:**
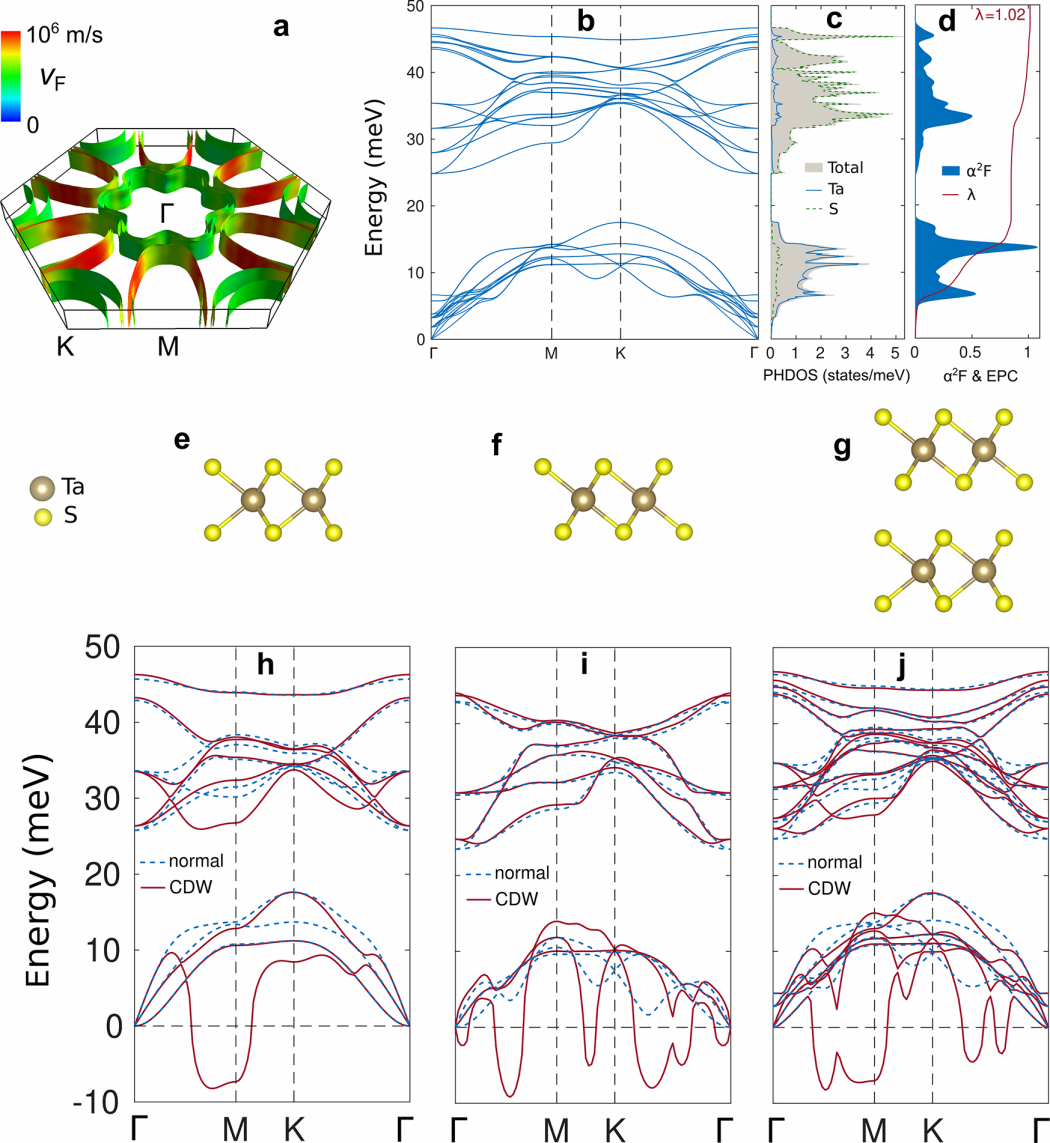
First-principles calculations of electronic and phononic properties of 6R TaS_2_. (a) Fermi surface of 6R TaS_2_, where the colors indicate the Fermi velocities. (b) Phonon band structure of 6R TaS_2_, (c) the corresponding total and atom-resolved phonon DOS (PHDOS), and (d) the Eliashberg function α^2^F and resulting electron–phonon coupling constant λ. Crystal structures of (e) ML 1H TaS_2_, (f) ML 1T TaS_2_, and (g) T–H heterobilayer. The corresponding phonon band structures in both the normal (dashed blue lines) and charge density wave (CDW) regimes (solid red lines) of (h) ML 1H TaS_2_, (i) ML 1T TaS_2_, and (j) T–H heterobilayer.

The close similarity between the 6R phase and the constituent 1T-1H BLs persists in our DFPT results on the phonon density of states (PHDOS) and α^2^F, the Eliashberg spectral function of the electron–phonon coupling (Figure 4c,d and Figure S16c,f). Moreover, we found the resulting electron–phonon coupling constant of the 6R phase (λ = 1.02, Figure 4d) to be very close to that of the 1T-1H BL (λ = 1.04, Figure S16f) but also to λ of the 1H ML (λ = 1.07, Figure S16d). This provides clear proof that 6R TaS_2_ indeed hosts superconductivity, which is in agreement with our magnetic and transport measurements. Moreover, these results indicate the superconducting phase of 6R TaS_2_ to be driven by the 1H planes.

As our experimental results have revealed a CDW state akin to that of bulk 1T TaS_2_ to occur in 6R TaS_2_, we set out to explore its microscopic origins through first-principles calculations. Both the 1H and 1T ML display CDW-type instabilities in their phonon dispersions, as shown in Figure 4h,i, which have resolved by lowering the broadening factor of the Fermi–Dirac smearing function for the electronic occupation (see Supporting Information for computational details). The phonon dispersion of the 1H ML (Figure 4h) shows an instability around M, leading to a simple integer lattice reconstruction (into an *n* × *n* supercell, with *n* a natural number ranging from 2 to 8 according to our DFPT result). On the other hand, the phonon dispersion of the 1T ML (Figure 4i) shows more complex behavior, as it relates to the √13 × √13 lattice reconstruction known as star-of-David. Through an analogous calculation on the 1T-1H BL, shown in Figure 4j, we found that both CDW types coexist in this system, albeit spatially separated in the respective layers.

Altogether, our first-principles results indicate that the 1T layers in 6R TaS_2_ will be insulating at temperatures well below the CCDW-*T*_c_ (305 K in 6R TaS_2_, Figure 3a) because of the Mott state. Thus, the occurrence of ML H-type TaS_2_, surrounded by insulating 1T layers, can explain the increase in superconducting *T*_c_ in our bulk 6R TaS_2_ sample, far exceeding the *T*_c_ of bulk 2H TaS_2_. Such enhancements were also noted for 2H TaS_2_ samples where the superconducting 2H layers were decoupled by intercalation.^34^

In conclusion, we have reported the phase transition of 1T TaS_2_ into 6R TaS_2_ when heated at 800 °C. The as-prepared 6R TaS_2_ shows the coexistence of superconductivity with a *T*_c_ of 2.6 K and CDW transitions at 320 and 305 K. Our TEM analysis shows the presence of a commensurate CDW phase at room temperature. Exfoliation and random restacking of layers in 6R TaS_2_ enhance the superconducting *T*_c_ to 3.6 K while decreasing the NC to CCDW transition to 250 K. The superconducting *T*_c_ and CDW transition temperatures closely resembles that of monolayer 1H and few-layer 1T TaS_2_ samples, respectively, indicating separation of 1T and 1H sheets in 6R TaS_2_. Our first-principles calculations show the coexistence of the main electronic, vibrational, and electron–phonon coupling properties of individual T and H layers in mixed T-H layers in 6R TaS_2_. This suggests that the origin of superconductivity in 6R TaS_2_ lies in the 1H layers of the crystal, separated from one another by insulating 1T layers in the CCDW state, scarcely interfering with the superconducting state. This alternating layered structure makes this material a true 2D superconductor in bulk form and opens a plethora of intriguing questions related to Josephson physics, THz radiation, and so forth. Further work is needed to understand the dynamics of reported phase transition in TaS_2_. This could enable the controlled synthesis of different polytypes of layered chalcogenide materials.

## Supporting Information Available

Materials and methods; preparation of 6R TaS_2_ and *in**situ* XRD characterization, electron microscopy, magnetic measurements, electrical transport measurements, preparation of exfoliated 6R TaS_2_, electrical measurements using mechanically exfoliated 6R TaS_2_, and first-principles calculations (PDF)
