# Supplementary material for: Alternating Superconducting and Charge Density Wave Monolayers within Bulk 6R-TaS2
Source: Nano Lett. 2022 Jul 20;22(15):6268–75. doi: 10.1021/acs.nanolett.2c01851 (PMC9373026; doi:10.1021/acs.nanolett.2c01851)
Supplement: Supplementary file 1 — nl2c01851_si_001.pdf [file nl2c01851_si_001.pdf]

Supporting Information

Alternating Superconducting and Charge Density Wave Monolayers within Bulk 6R-TaS_2

Amritroop Achari[1,2], Jonas Bekaert[3,4], Vishnu Sreepal[1,2], Andrey Orekhov[4,5], Piranavan. Kumaravadivel[1,6], Minsoo Kim[6], Nicolas Gauquelin[4,5], Premlal Balakrishna Pillai[1,2], Johan Verbeeck[4,5], Francois M. Peeters[3], Andre K. Geim[1,6], Milorad V. Milošević[3,4], Rahul R. Nair[1,2*]*

[1]National Graphene Institute, University of Manchester, Manchester, M13 9PL, UK.

[2]Department of Chemical Engineering, University of Manchester, Manchester, M13 9PL, UK.

[3]Department of Physics, University of Antwerp, Groenenborgerlaan 171, B-2020, Antwerp, Belgium.

[4]NANOlab Center of Excellence, University of Antwerp, Groenenborgerlaan 171, B-2020 Antwerp, Belgium.

[5]Electron Microscopy for Materials Science (EMAT), University of Antwerp, Groenenborgerlaan 171, B-2020 Antwerp, Belgium.

[6]Department of Physics and Astronomy, University of Manchester, Manchester, M13 9PL, UK.

*Corresponding author. Email: amritroop.achari@manchester.ac.uk

rahul@manchester.ac.uk, Tel: +44 (0) 1613066574

Materials and Methods

Preparation of 6R TaS_2 and *in situ* XRD characterisation: Single crystalline 1T and 2H TaS_2 crystals were obtained from HQ graphene and used as such. A piece of 1T TaS_2 crystal (dimension approx. 2 mm x 2 mm) was cleaved from the single crystal and placed on a heating stage of Rigaku diffractometer with a graphite dome. The crystal was heated under vacuum (approx. 10^{-3} bar) at 800 °C for 5 h to convert it into 6R form.

Phase transition in the 1T TaS_2 crystals was probed by *in situ* X-ray diffraction studies using a Rigaku Smartlab XRD system (Cu Kα, λ=0.15406 nm). A small piece (4 x 4 mm) of 1T TaS_2 sample was placed on the heating stage of the XRD instrument. The position and height of the sample were carefully aligned so that the sample was properly exposed to the X-ray beam. A Kβ filter was used to remove extra peaks from the Cu Kβ wavelength. The heating stage containing the sample was fitted with a graphite dome and a vacuum of 10^{-3} mbar was applied. The sample was heated at 30 °C/min and spectra were collected at regular temperature intervals. The sample was stabilised at each temperature before XRD data was collected.

Powder XRD data of the samples were collected using a D-8 Discover advanced XRD system (Cu Kα, λ=0.15406 nm). The samples were ground in a mortar before performing the powder XRD.

Raman spectra was measured with HORIBA Raman spectrometer (XploRA PLUS) with a laser excitation of 532 nm (spot size ~1 μm, laser power of 0.125 mW and spectrometer grating of 1200 grooves per millimeter).

Electron microscopy: A state-of-the-art double corrected Thermo Fisher Titan QU-Ant-EM transmission electron microscope was used for TEM imaging. High-resolution HAADF-STEM

images were acquired at 300 kV using a convergence semi-angle α of 21 mrad, 50 pA probe current and Cs tuned close to 0 µm. To minimize distortions during long scanning in STEM mode, a time series technique was employed.

In situ cooling experiments were performed on a Thermo Fisher Osiris transmission electron microscope operated at 200 kV. Temperature experiments were performed using a Gatan 636 double-tilt liquid nitrogen cooling holder.

Cross-sectional TEM foils were prepared using FIB from bulk 1T and 6R TaS_2 samples using the lift out method in a Thermo Fisher dual beam FIB/SEM instrument. A protective platinum layer using electron beam assisted deposition has been used. An ion beam of 2 kV/0.2 nA was employed to achieve the final thinning and to minimize defects generated during high voltage FIB thinning on both sides of the sample.

TEM data were processed with custom-made scripts based on the open-source python libraries *Hyperspy*[1] and *Pixstem*[2].

Magnetic measurements: A Quantum Design Magnetic Property Measurement System (MPMS-3) was used to measure the temperature- and field-dependent magnetization of the samples. In the zero-field cooling (ZFC) mode, the samples were initially cooled to 1.8 K in zero applied field, then a desired external field H was applied and the magnetisation M was measured as a function of increasing temperature, T (typically 1.8–20 K). The field-cooling (FC) part of an $M(T)$ curve was obtained on cooling the sample to 1.8 K in the same H.

The magnetisation measurements were performed in two different geometries. For in-plane measurements, samples were mounted on a quartz rod with GE varnish keeping the c-axis perpendicular to the direction of the magnetic field. Out of plane measurements were done by placing the crystal at the bottom of a gelatine capsule and mounting it in the magnetometer

using a straw sample holder in such a way that the c-axis of the crystal was parallel to the magnetic field direction. Since both sample holders had a negligible magnetic response, the magnetic data was used without any background correction.

Electrical transport measurements: For electrical transport measurements, we have fabricated TaS_2 devices in a linear four-probe geometry. Four contacts were made with silver paint on a 2 × 4 mm rectangle sample of a TaS_2 crystal. The samples were loaded on an Electrical Transport Option (ETO) sample holder and sample rod in MPMS-3. The ETO is capable of measuring differential resistance as a function of current or voltage. This is achieved by applying a small AC excitation on top of a DC offset bias. The AC response is measured and used to calculate the differential resistance. The differential resistance is a direct measure of the first derivative of the IV curve at a given DC bias.

Preparation of exfoliated 6R TaS_2: Exfoliated samples of 6R TaS_2 were prepared by lithium intercalation of TaS_2 followed by exfoliation. Lithium intercalation of the crystals was done inside a glove box. In a typical procedure, a small piece of 6R TaS_2 crystal (approx. 5 mg) was immersed in 0.1 mL of 1.6 M solution of n-butyl lithium in a glass vial. The vial was rested for 48 hours before washing the crystal with hexane three times. The lithium intercalated crystal was dried and used for liquid-phase exfoliation of 6R TaS_2. Liquid phase exfoliation was performed by sonicating lithium intercalated 6R TaS_2 in water for 1 h to exfoliate into flakes. Such exfoliated samples were unstable in water and prone to oxidation if stored in water for longer than 24 hours. The dispersion was quickly centrifuged at 12000 rpm, followed by washing 3 times with water and 1 time with ethanol. The washed sample was dried under a vacuum for magnetic measurements.

Electrical measurements using mechanically exfoliated 6R TaS_2: The TaS_2 layers were exfoliated from bulk crystals onto SiO_2/Si substrates in an argon-filled glovebox with levels of

O_2 and H_2O below 0.5 ppm. Thin layers of TaS_2 (~ 1nm) were identified by using optical contrast under an optical microscope. Since mechanically exfoliated thin layers of TaS_2 are prone to oxidation, to prevent any exposure of TaS_2 to air during measurements, thin hexagonal boron nitride crystal was used as the capping layer and few-layer graphite strips as contact electrodes. They were aligned and transferred onto the monolayer in the Ar-filled glovebox using the standard dry transfer process with polypropylene carbonate (PPC) coated polydimethylsiloxane (PDMS) films as stamps. Later, 1D metal contacts to the graphene strips were fabricated by standard methods as reported in our previous work[3].

The critical temperature of thin TaS_2 (outlined by the red dotted line in Figure S14a) was measured using exfoliated strips of graphene as the contact electrodes (outlined by the black dotted lines in Figure S14a). The current-voltage (*I-V*) characteristics of the device were measured by applying the small AC excitation current of 10 nA and sweeping the DC bias current (Figure S14b). While the electric current was applied across the flake of 6R-TaS_2 through the graphene electrodes, voltage is measured on the graphene electrode that is only in contact with the few-layer 6R-TaS_2. This is to avoid any contribution from multilayer TaS_2 that is in contact with the other electrode (see Figure S14a). Dips in the measured differential resistance (*dV/dI*) are signatures of superconductivity. Since the contact between the graphene electrode and TaS_2 is not transparent, *dV/dI* increases near zero bias. By carefully examining the temperature dependence of *dV/dI* as a function of DC biasing current (Figure S14c), T_c is determined to be ~ 2.4 K, where the non-linear *I-V* characteristics due to superconductivity disappear.

First-principles calculations: Our density functional theory (DFT) calculations make use of the Perdew-Burke-Ernzerhof (PBE) functional implemented within the ABINIT code[4]. We included spin-orbit coupling (SOC) using fully relativistic Goedecker pseudopotentials[5,6].

Here, Ta-$5d^3 6s^2$ and S-$3s^2 3p^4$ states were included as valence electrons, together with an energy cutoff of 50 Ha for the plane-wave basis. Van der Waals interactions were included via the vdw-DFT-D3 method introduced by Grimme,[7] based on the Becke-Jonhson formalism[8]. All crystal structures were relaxed so that forces on each atom were below 1 meV/Å. To simulate the 2D structures (ML 1T and 1H and the 1T-1H BL), 25 Å of vacuum was included in the unit cells. In all density functional perturbation theory (DFPT) calculations, a 24 × 24 × 1 k-point grid and a 12 × 12 × 1 q-point grid were employed. SOC was omitted in the DFPT calculation on 6R TaS_2, due to computational restrictions posed by the size of its unit cell.

To identify CDWs, we have compared DFPT results obtained with higher (0.01 Ha) and lower (0.0025 Ha) broadening factors of the Fermi-Dirac smearing function for the electronic occupations. The case with lower broadening resolves the phonon instabilities corresponding to the CDW (Figure 5). To evaluate the electron-phonon coupling (EPC) responsible for the superconducting state, we have used Migdal-Eliashberg theory[9], based on the EPC matrix elements obtained through the DFPT calculations.

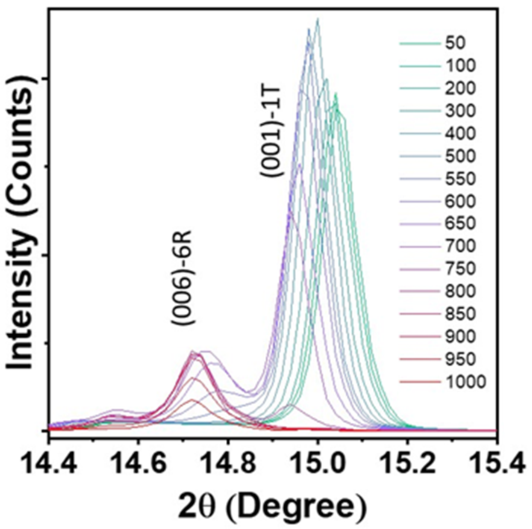

Figure S1. *In situ* XRD showing the transition of a single crystalline 1T TaS_2 into 6R phase. Phase conversion of the 1T TaS_2 samples was measured *in situ* on a heated XRD stage at regular temperature intervals as shown in the figure. At temperatures below 500 °C, we only observed a monotonous downshifting of (001) peak associated with the thermal expansion of the crystalline *c* axis. Above 600 °C we noticed the appearance of a new peak at lower 2θ (14.78°) compared to the original peak of the 1T phase at 15.05°.

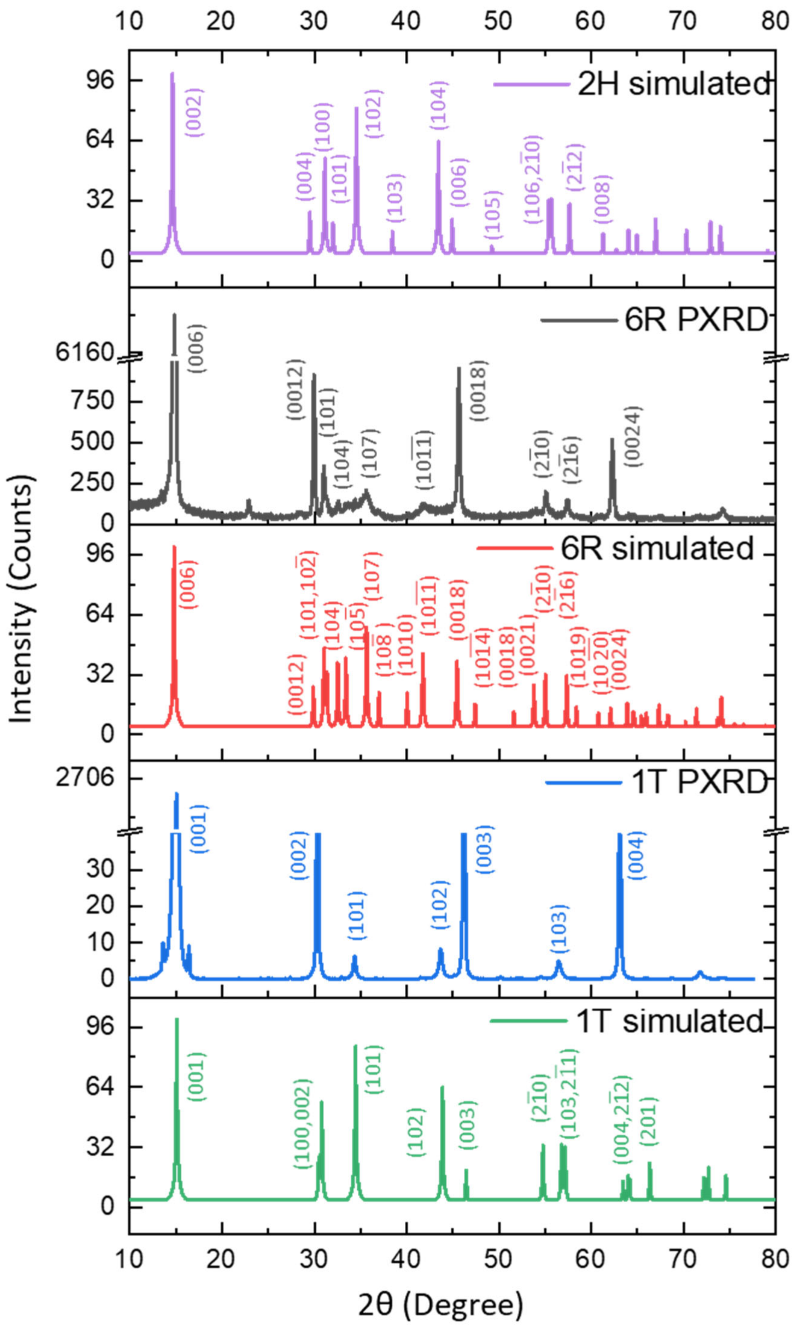

Figure S2. Powder XRD of 6R and 1T TaS_2. Comparison of powder XRD pattern of 6R TaS_2 with that of 1T, and simulated PXRD patterns of 1T, 2H and 6R phases. The PXRD pattern of 6R TaS_2 shows peaks matching with (104), (107), $(10\overline{11})$ peaks in the simulated 6R phase, which are not present in 1T or 2H TaS_2.

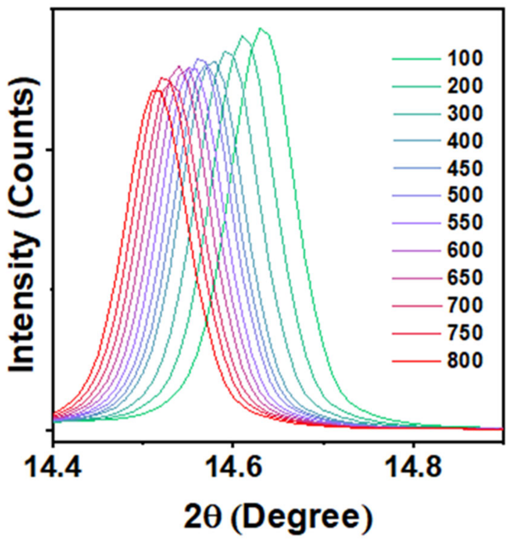

Figure S3. *In situ* XRD during annealing of 2H TaS_2. Temperature-dependent XRD of 2H TaS_2 heated up to 800 °C showing the monotonous shift in the (002) peak. 2H being the most stable phase among the TaS_2 polytypes, we do not see any transition into the 6R phase up to 800 °C. The downshifting of the peak is due to the thermal expansion of the crystal along the c-axis. After cooling, the peak reverts to its original position.

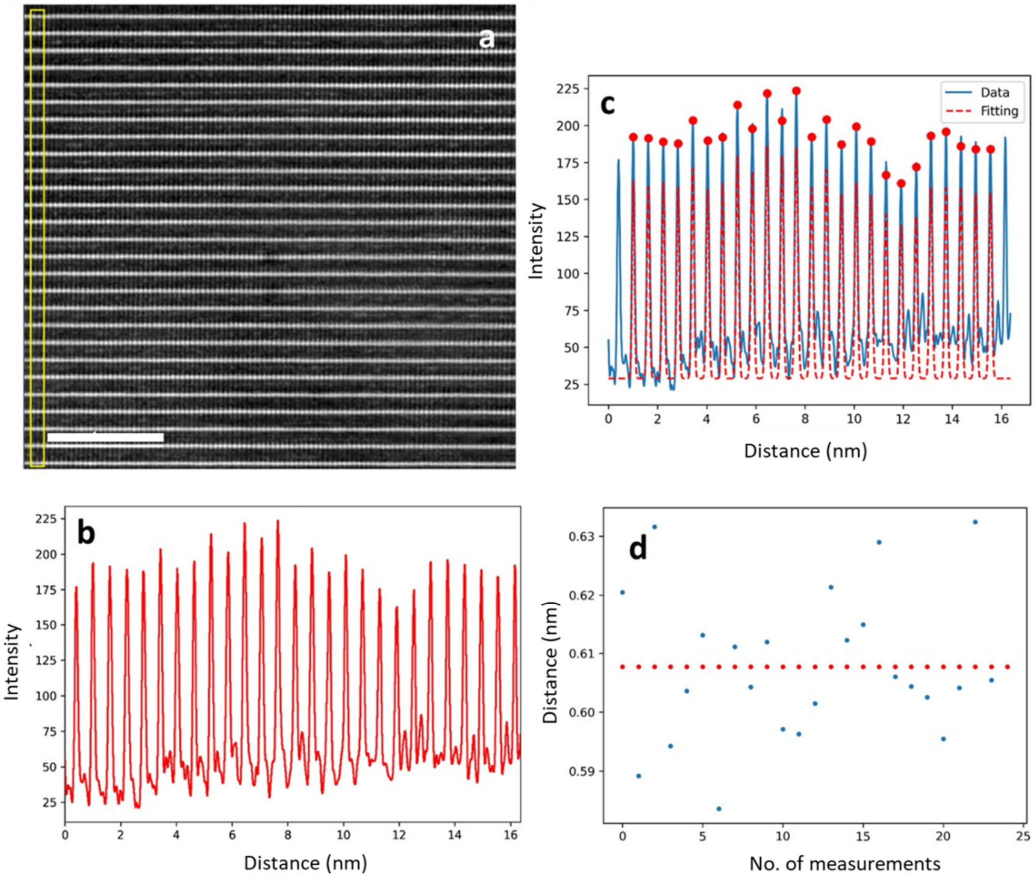

Figure S4. Analysis of HRSTEM data. (a) HRSTEM image of 6R TaS_2 cross-section showing an example rectangle section along which intensity profiles were estimated. (Scale bar, 4 nm). The average from five such regions was plotted in (b). The red dotted lines in figure (c) correspond to the fitted Gaussian function for every intensity profile in (b). The red circles represent the centre of the Gaussians. (d) d-spacing (blue dots) and it's mean (red dotted line) along (00l) TaS_2 direction are plotted according to the position of the centre Gaussians (red circles in Figure c).

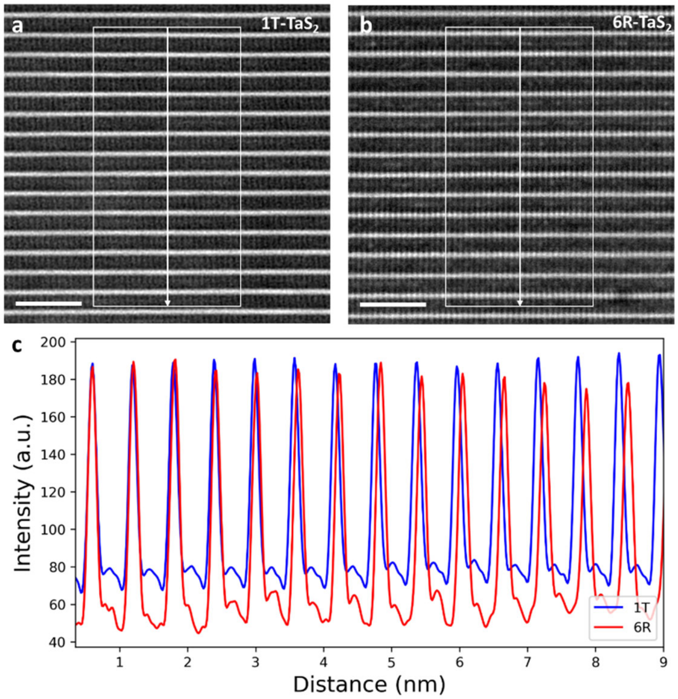

Figure S5. Lattice expansion in 6R TaS_2. High-resolution STEM images of the pristine 1T (a) and after the heat treatment at 800 °C in vacuum (b). Scale bar, 2nm (c) Comparison of the intensity profiles along (0001) direction taken from the rectangular regions in a, b shows an increase in interlayer spacing after heating.

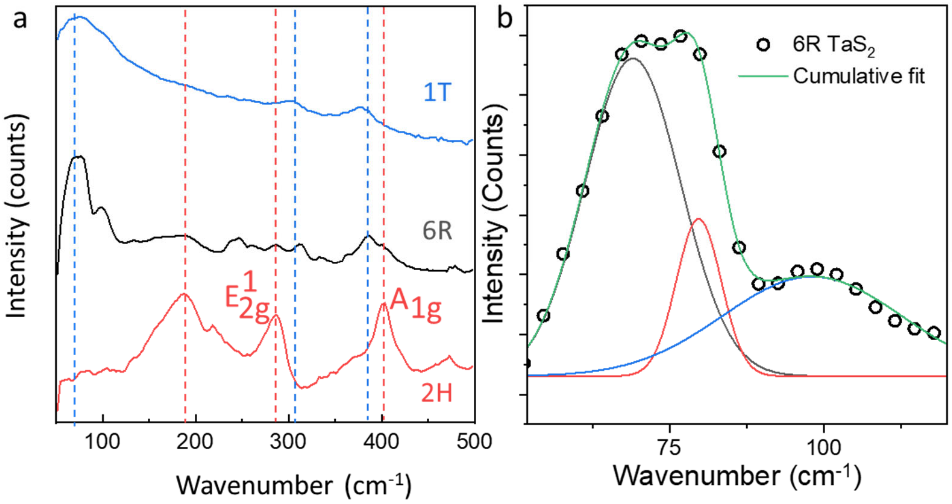

Figure S6. Raman spectra of 6R, 1T, and 2H TaS_2. (a) Comparison of Raman spectra between 1T, 2H and 6R TaS_2 samples showing the presence of features from both 1T and 2H phase in the 6R sample. The 2H sample shows typical E_{2g}^{1} and A_{1g} Raman modes at 280 and 400 cm^{-1} respectively[10]. The peak at 180 cm^{-1} is observed due to second order scattering[11]. All of these peaks appear in the 6R sample. (Red dotted lines) On the other hand, the peaks observed for bulk 1T TaS_2 at 80, 303 and 384 cm^{-1} corresponding to the nearly commensurate structure at room temperature[12] are also present in the 6R sample (Blue dotted lines) but with additional features. Interestingly, the low frequency peak of 6R sample becomes sharper and well defined compared to the 1T phase with the appearance of a peak at 243 cm^{-1}, signifying the presence of commensurate structure[13]. (b) Enlarged view of the low frequency modes in 6R TaS_2 showing deconvoluted peaks at 70, 80 and 98 cm^{-1}, these phonon bands originate from folding of the Brillouin zone signifying CCDW phase transition in the 1T layers of 6R TaS_2 at room temperature.[14]

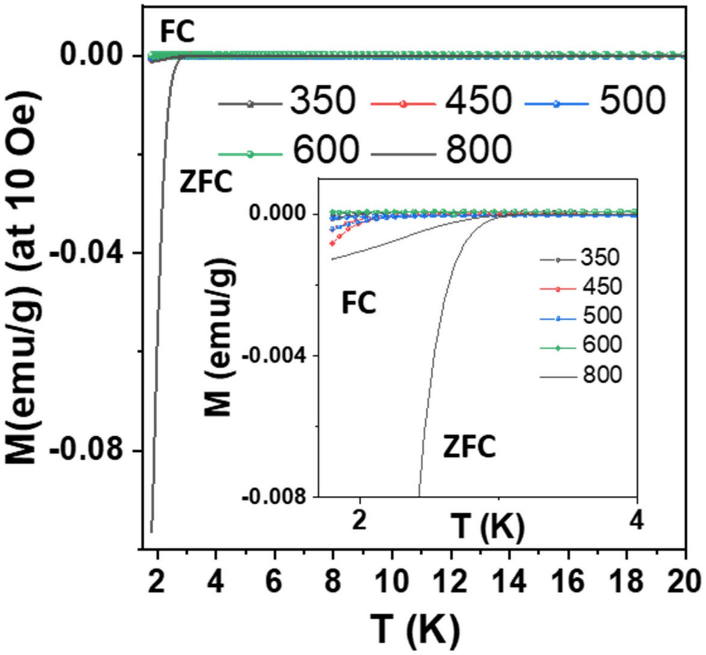

Figure S7. 1T to 6R transition seen in magnetisation measurements. Temperature dependence of ZFC and FC magnetisation, M, for single-crystalline1T TaS_2 heated at different temperatures under vacuum at 10 Oe magnetic field. Inset: zoomed in magnetic transition.

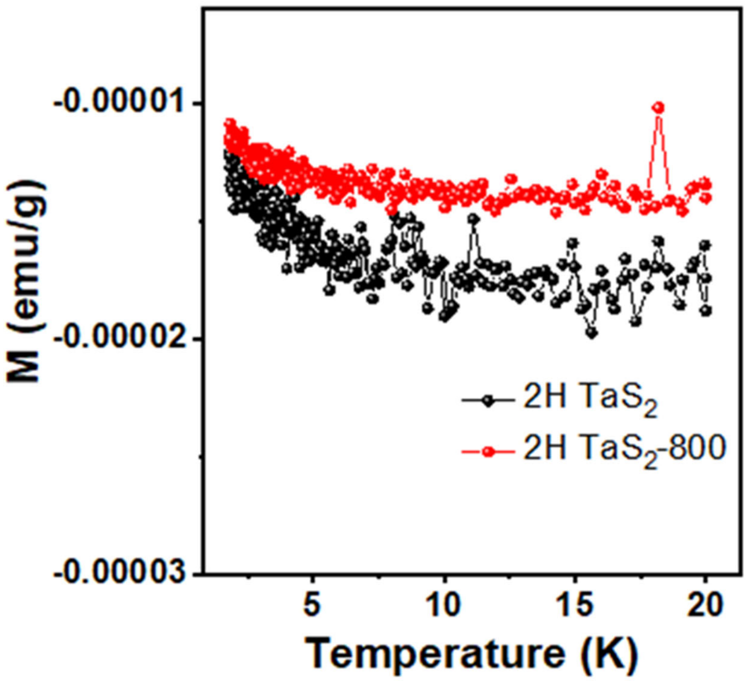

Figure S8. Low temperature magnetisation of 2H TaS_2 crystal before and after heating. ZFC and FC temperature-dependent magnetisation of 2H TaS_2 and 2H TaS_2 heated at 800 °C under vacuum, showing no sign of superconductivity down to 1.8 K.

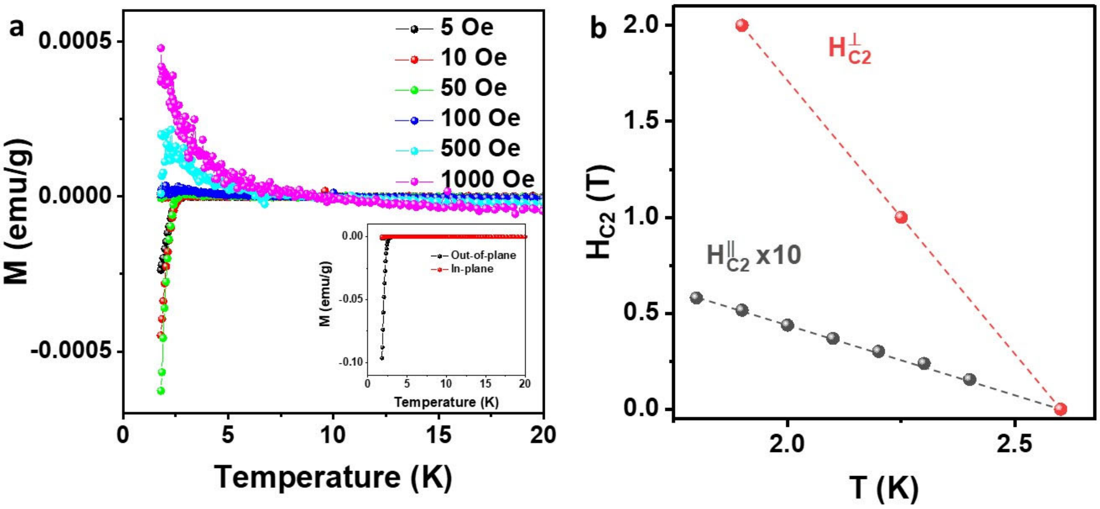

Figure S9. Anisotropic superconductivity in 6R TaS_2. a) ZFC and FC temperature-dependent magnetisation of 6R TaS_2 with its *c*-axis perpendicular to the magnetic field. Inset: Comparison of in-plane ($ab \parallel H$) and out-of-plane ($c \parallel H$) magnetisation measurement carried out at 10 Oe. b) H_{C2} as a function of temperature for in-plane (*c*-axis perpendicular to the applied field, $H_{C2}^{\perp}$, red spheres) and out-of-plane (*c*-axis parallel to the applied field, $H_{C2}^{\parallel}$, black spheres) superconductivity. For better visibility $H_{C2}^{\parallel}$ has been multiplied by 10.

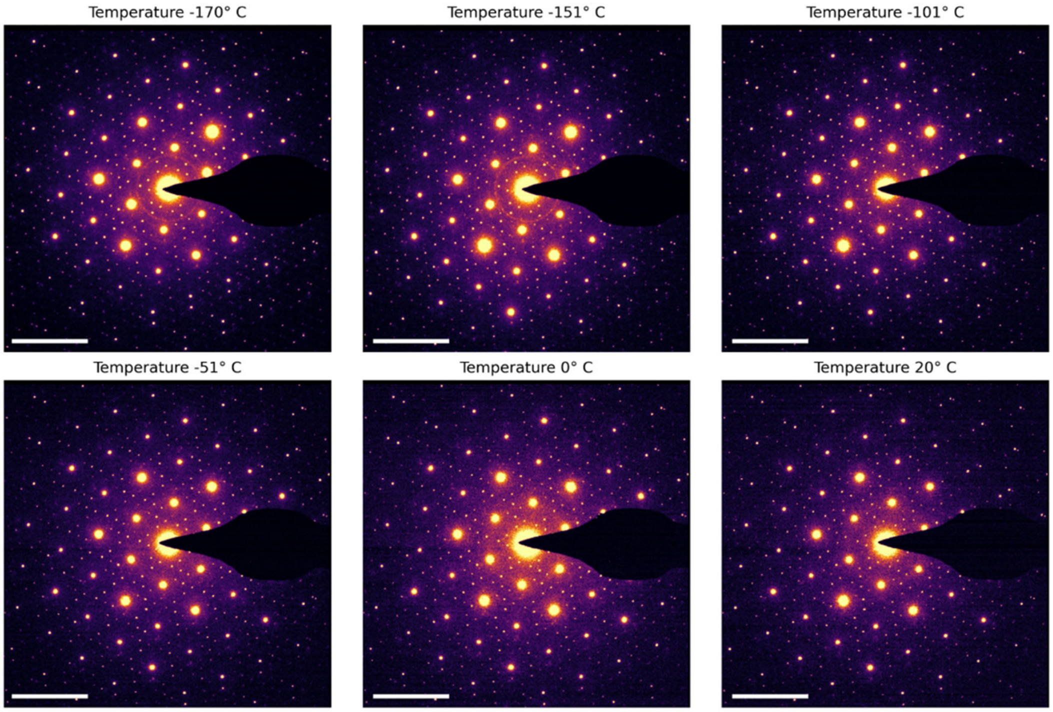

Figure S10. Low temperature electron diffraction. Selected area electron diffraction patterns acquired during the *in situ* cooling experiment in the range (-170 °C to 20 °C). No additional diffractions spots were noticed while cooling the sample. Scale bar, 6 1/nm.

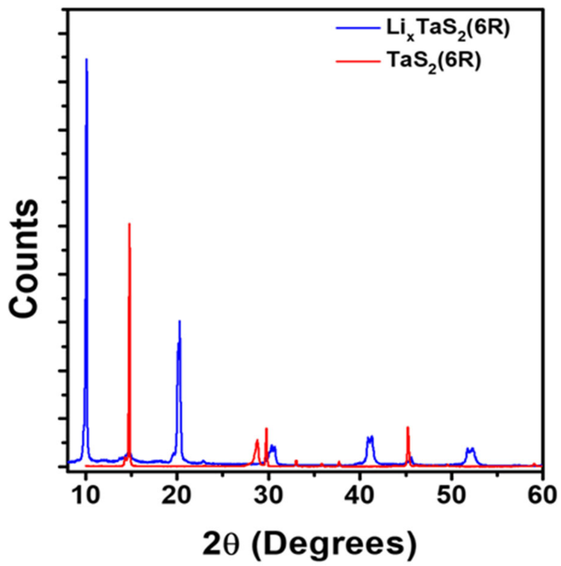

Figure S11. Lithiation of 6R TaS_2. XRD pattern of 6R TaS_2 and lithiated 6R TaS_2 measured on single crystalline samples. The crystals are oriented on their *ab* plane; hence only 00l peaks appear in the XRD. From the XRD spectra, the low angle peak (006) of the 6R TaS_2 shifts from 14.8° to 10° after lithiation, indicating the intercalation of lithium into the interlayer spaces. The interlayer spacing for the intercalated sample was 0.873 nm compared to 6R TaS_2 with a d spacing of 0.598 nm. It is to be noted that the lithiated sample was prone to oxidation in air and hence was stored in a glove box under an inert atmosphere.

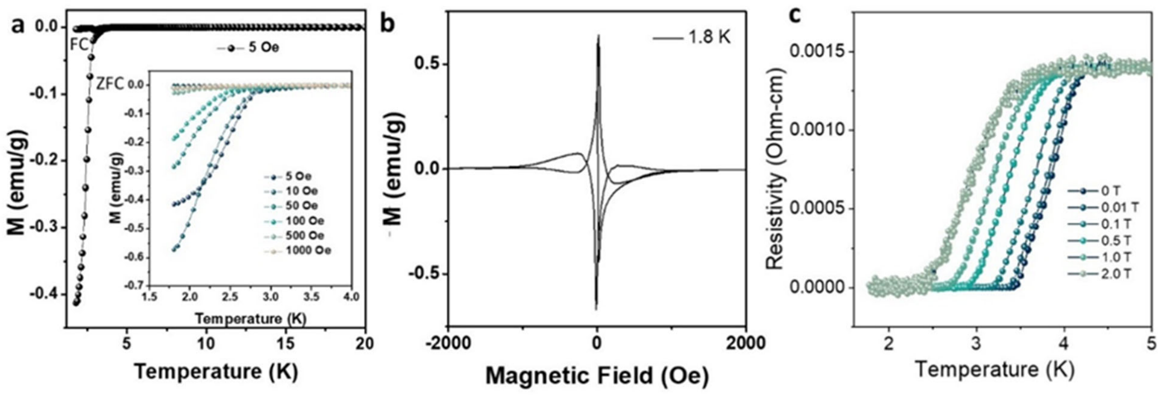

Figure S12. Superconductivity in lithiated 6R TaS_2. (a) Temperature dependence of magnetisation of single-crystalline 6R TaS_2 intercalated with lithium under the magnetic field of 5 Oe, with zero-field-cooling curve and field-cooling curve. Inset shows field dependence of transition temperature of lithiated 6R TaS_2. We observed an increase in transition temperature from 2.6 K in as-prepared 6R TaS_2 to 3.0 K when lithium was introduced in the system. (b) Magnetic hysteresis of lithium intercalated 6R TaS_2 at 1.8 K, showing type 2 superconducting nature. (c) Field dependence of electrical resistance of lithiated 6R TaS_2 showing superconducting transition at ~ 3K.

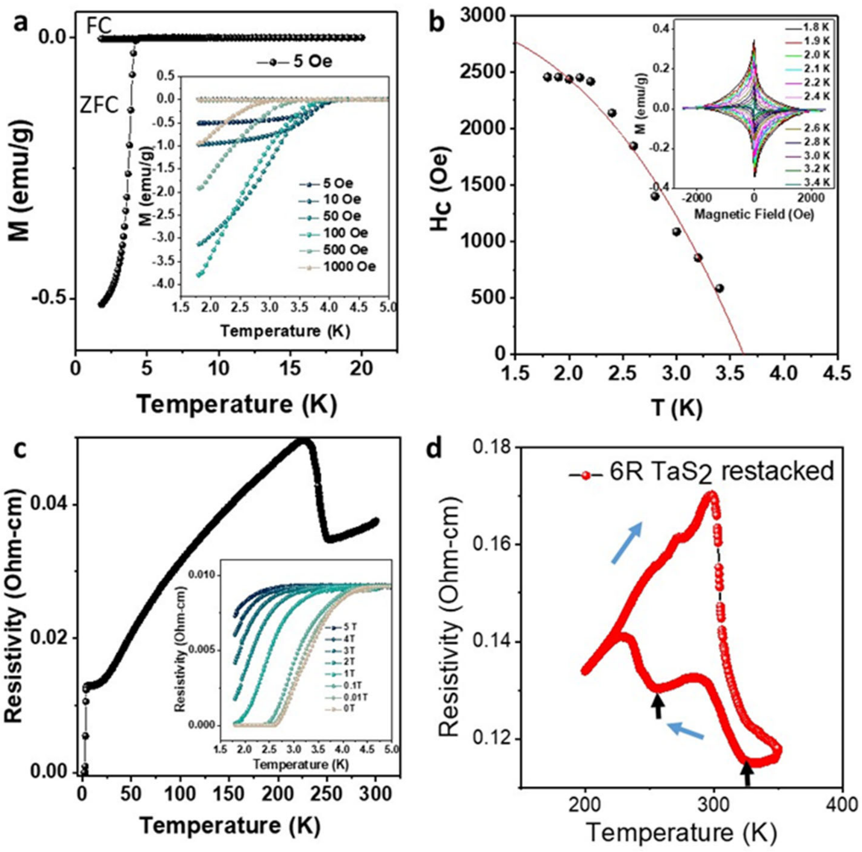

Figure S13. Superconductivity in restacked 6R TaS_2. (a) Temperature dependence of magnetisation of restacked 6R TaS_2 under the magnetic field of 5 Oe, with zero-field-cooling curve and field-cooling curve. Inset shows field dependence of transition temperature of restacked 6R TaS_2. (b) Temperature dependence of upper critical field Hc_2. The upper critical field was calculated from the divergence point in the M-H hysteresis curves (inset). The red line represents the best least squares fit of the equation $H_{C2}(T) = H_{C2}[1-(T/T_C)^{(1+\alpha)}]$, where H_{C2} and α are the fitting variables[15]. (c) Temperature dependence of electrical resistivity of restacked 6R TaS_2 at H = 0 T. inset: Field dependence of electrical resistance of restacked 6R TaS_2 (d) Temperature-dependence of electrical resistivity of restacked 6R TaS_2 showing CDW transitions at 250 K and 320 K from nearly commensurate to commensurate and incommensurate to nearly commensurate phases respectively (marked by black arrows). Blue arrows denote temperature sweep direction. It is to be noted that the restacked samples

were not stable in air for more than 24 hours; hence we performed all measurements immediately after preparing the samples.

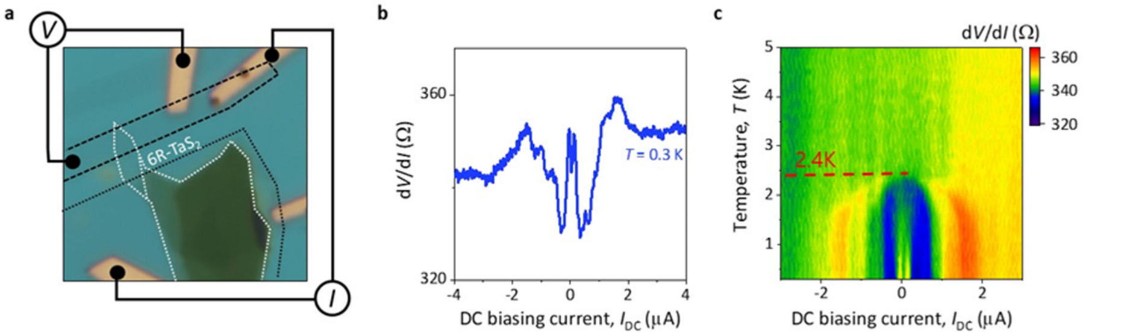

Figure S14. Electron transport measurements of mechanically exfoliated TaS_2. (a) Optical micrograph of few-layer 6R-TaS_2 device with schematics of the wiring used for measurements. The black dotted line outlines the thin graphite electrodes and the white dotted line outlines the TaS_2 flakes. The entire TaS_2 and graphite electrodes were capped by a thin layer of hexagonal boron nitride crystal. (b) Measured differential resistance (*dV/dI*) as a function of DC biasing current (I_{DC})at temperature T= 0.3 K. (c) *dV/dI* as a function of I_{DC} and temperature T. The red dotted line indicates the superconducting transition temperature, above which the non-linear *I*-*V* characteristics disappear.

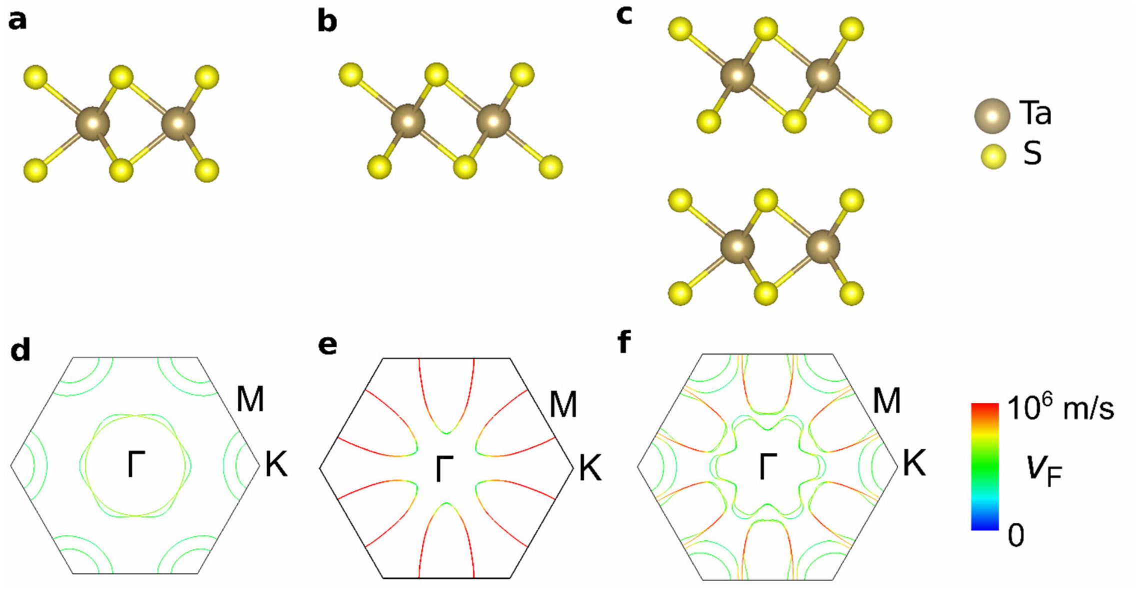

Figure S15. Fermi surfaces of monolayer and bilayer TaS_2. Crystal structure of (a) ML 1H TaS_2, (b) ML 1T TaS_2, and (c) BL 1T-1H TaS_2. Fermi surface of (d) ML 1H TaS_2, (e) ML 1T TaS_2 and (f) BL 1T-1H TaS_2, where the colours indicate the Fermi velocities.

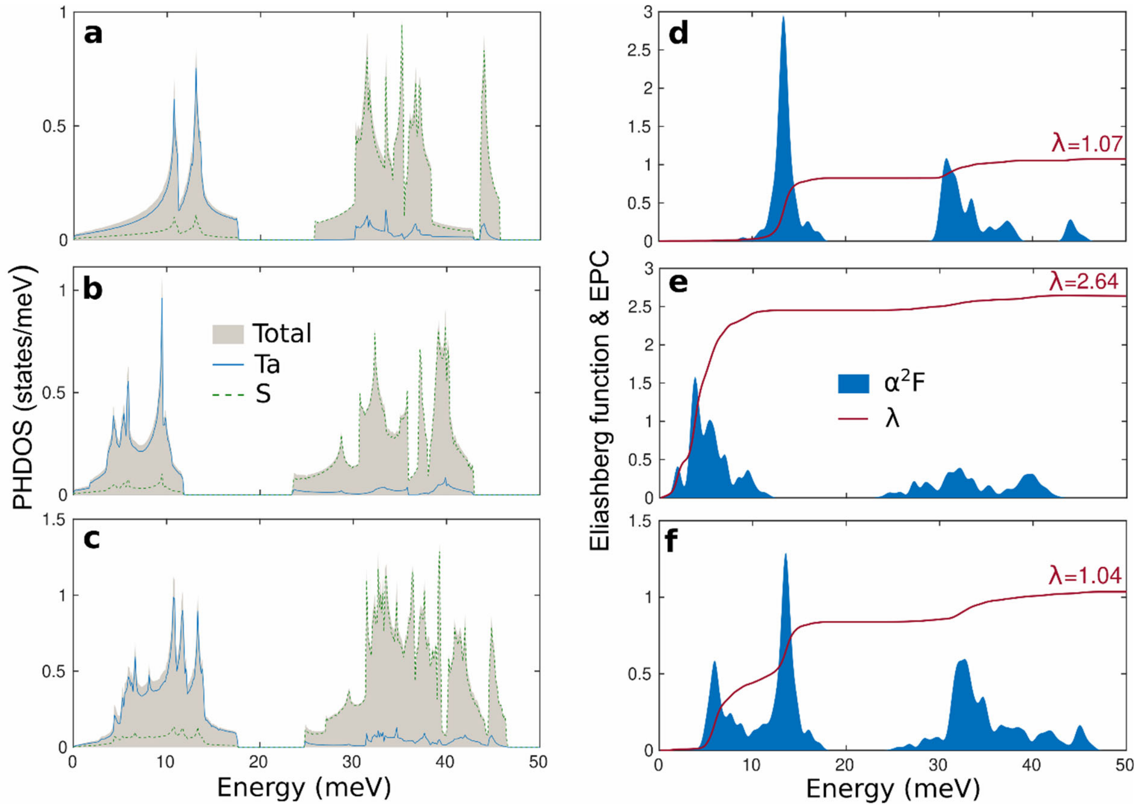

Figure S16. Phonon density of states and electron-phonon coupling in monolayer and bilayer TaS_2. Total and atom-resolved phonon density of states (PHDOS) of (a) ML 1H TaS_2, (b) ML 1T TaS_2 and (c) BL 1T-1H TaS_2, and Eliashberg function (α^2F) and electron-phonon coupling (EPC, λ) of (d) ML 1H TaS_2, (e) ML 1T TaS_2 and (f) BL 1T-1H TaS_2.

References:

1. de la Peña, F.; Prestat, E.; Tonaas Fauske, V.; Burdet, P.; Jokubauskas, P.; Nord, M.; Ostasevicius, T.; MacArthur, K. E.; Sarahan, M.; Johnstone, D. N., hyperspy/hyperspy: HyperSpy v1. 5.2. *zndo* **2019**.
2. Nord, M.; Webster, R. W. H.; Paton, K. A.; McVitie, S.; McGrouther, D.; MacLaren, I.; Paterson, G. W., Fast Pixelated Detectors in Scanning Transmission Electron Microscopy. Part I: Data Acquisition, Live Processing, and Storage. *Microsc. Microanal.* **2020,** *26* (4), 653-666.
3. Kim, M.; Kumaravadivel, P.; Birkbeck, J.; Kuang, W.; Xu, S. G.; Hopkinson, D. G.; Knolle, J.; McClarty, P. A.; Berdyugin, A. I.; Ben Shalom, M.; Gorbachev, R. V.; Haigh, S. J.; Liu, S.; Edgar, J. H.; Novoselov, K. S.; Grigorieva, I. V.; Geim, A. K., Micromagnetometry of two-dimensional ferromagnets. *Nat. Elelctron.* **2019,** *2* (10), 457-463.
4. Gonze, X. *et al.* , The Abinitproject: Impact, environment and recent developments. *Comput. Phys. Commun.* **2020,** *248*, 107042.
5. Goedecker, S.; Teter, M.; Hutter, J., Separable dual-space Gaussian pseudopotentials. *Phys. Rev. B* **1996,** *54* (3), 1703-1710.

6. Krack, M., Pseudopotentials for H to Kr optimized for gradient-corrected exchange-correlation functionals. *Theor. Chem. Acc.* **2005,** *114* (1), 145-152.
7. Grimme, S.; Antony, J.; Ehrlich, S.; Krieg, H., A consistent and accurate ab initio parametrization of density functional dispersion correction (DFT-D) for the 94 elements H-Pu. *J. Chem. Phys.* **2010,** *132* (15), 154104.
8. Becke, A. D.; Johnson, E. R., A simple effective potential for exchange. *J. Chem. Phys.* **2006,** *124* (22), 221101.
9. Eliashberg, G., Temperature Green's function for electrons in a superconductor. *Sov. Phys. JETP* **1961,** *12* (5), 1000-1002.
10. Sugai, S.; Murase, K.; Uchida, S.; Tanaka, S., Studies of lattice dynamics in 2H TaS_2 by Raman scattering. *Solid State Commun.* **1981,** *40* (4), 399-401.
11. Liu, W.; Duan, Z.; Zhang, C.; Hu, X. X.; Cao, J. B.; Liu, L. J.; Lin, L., Experimental observations and density functional simulations on the structural transition behavior of a two-dimensional transition-metal dichalcogenide. *Sci. Rep.* **2020,** *10* (1), 18255.
12. Duffey, J. R.; Kirby, R. D.; Coleman, R. V., Raman scattering from 1T-TaS_2. *Solid State Commun.* **1976,** *20* (6), 617-621.
13. Hirata, T.; Ohuchi, F. S., Temperature dependence of the Raman spectra of 1T-TaS_2. *Solid State Commun.* **2001,** *117* (6), 361-364.
14. Lacinska, E. M.; Furman, M.; Binder, J.; Lutsyk, I.; Kowalczyk, P. J.; Stepniewski, R.; Wysmolek, A., Raman Optical Activity of 1T-TaS_2. *Nano Lett.* **2022,** *22* (7), 2835-2842.
15. Qi, Y.; Naumov, P. G.; Ali, M. N.; Rajamathi, C. R.; Schnelle, W.; Barkalov, O.; Hanfland, M.; Wu, S.-C.; Shekhar, C.; Sun, Y.; Süß, V.; Schmidt, M.; Schwarz, U.; Pippel, E.; Werner, P.; Hillebrand, R.; Förster, T.; Kampert, E.; Parkin, S.; Cava, R. J.; Felser, C.; Yan, B.; Medvedev, S. A., Superconductivity in Weyl semimetal candidate $MoTe_2$. *Nat. Commun.* **2016,** *7* (1), 11038.
